# Behavioral and electrophysiological auditory processing measures in traumatic brain injury after acoustically controlled auditory training: a long-term study

**DOI:** 10.1590/S1679-45082015AO3379

**Published:** 2015

**Authors:** Carolina Calsolari Figueiredo, Adriana Neves de Andrade, Andréa Tortosa Marangoni-Castan, Daniela Gil, Italo Capraro Suriano

**Affiliations:** 1 Universidade Federal de São Paulo, São Paulo, SP, Brazil.

**Keywords:** Neuronal plasticity, Auditory perceptual disorders, Learning, Acoustic stimulation, Brain injuries/complications

## Abstract

**Objective:**

To investigate the long-term efficacy of acoustically controlled auditory training in adults after tarumatic brain injury.

**Methods:**

A total of six audioogically normal individuals aged between 20 and 37 years were studied. They suffered severe traumatic brain injury with diffuse axional lesion and underwent an acoustically controlled auditory training program approximately one year before. The results obtained in the behavioral and electrophysiological evaluation of auditory processing immediately after acoustically controlled auditory training were compared to reassessment findings, one year later.

**Results:**

Quantitative analysis of auditory brainsteim response showed increased absolute latency of all waves and interpeak intervals, bilaterraly, when comparing both evaluations. Moreover, increased amplitude of all waves, and the wave V amplitude was statistically significant for the right ear, and wave III for the left ear. As to P3, decreased latency and increased amplitude were found for both ears in reassessment. The previous and current behavioral assessment showed similar results, except for the staggered spondaic words in the left ear and the amount of errors on the dichotic consonant-vowel test.

**Conclusion:**

The acoustically controlled auditory training was effective in the long run, since better latency and amplitude results were observed in the electrophysiological evaluation, in addition to stability of behavioral measures after one-year training.

## INTRODUCTION

Traumatic brain injury (TBI) is defined as any traumatic insult resulting in anatomic lesion or functional impairment of the meninges, brain or its vessels.^(1)^

The deformation of the brain due to the acceleration and deceleration causes primary lesions that can affect the neuronal substrate responsible for hearing and it may result in* déficit *central hearing impairment involving cortical and subortical auditory areas. Thus, individuals who suffer TBI can present an auditory processing disorder (APD), which can be identified by electrophysiological and behavioral tests.^(2)^

The acoustically controled auditory training (ACAT) is a set of conditions and/or acoustic tasks that are controled and modeled at will, aiming to maximize the plasticity effects of the central nervous system and other related systems, so that their neural bases and auditory behaviors can be positively altered.^(2,3)^

Acoustically controled auditory training can be used to habilitate or rehabilite the auditory skills that help the linguistic and phonemic processings required for speech comprehension and effective communicative functions.

In a literature review we found only two studies conducted with individuals who sustained TBI treated with ACAT, and both showed that the training could effectively result in neuronal plasticity through auditory stimulation.^(4,5)^ However, retesting was done right after the stimulation sessions without a longitudinal follow-up of the patients.

## OBJECTIVE

To investigate the long-term efficacy of the accoustically controled auditory training in adults who sustained traumatic brain injury.

## METHODS

The research was conducted at the auditory processing and electrophysiology laboratories of the Hearing Disorder Course at the *Universidade Federal de São Paulo* and approved by the Institutional Review Board of the same university, under number 0389/10. All subjects signed an Informed Consent Form.

The six participants were male, aged 20 to 37 years. They all presented diffuse axonal injury, two did not present any associated focal injury, two had subdural hematoma, one presented a temporal contusion, and one presented a subdural hematoma and temporal contusion.

The inclusion criteria were severe blunt TBI (Glasgow scale from 3 to 8 upon hospital admission), induced coma, diffuse axonal injury, time between injury and participation in the study ranging from 3 to 24 months, age between 18 and 50 years, right-handedness, complete High School Education, auditory thresholds within the normal range between 250 and 4,000Hz.

All individuals had undergone an ACAT program approximately one year before. Results of the behavioral and electrophysiological tests performed right after the ACAT and one year after the training were compared.

The auditory evoked potentials (AEP) recording started with the long latency auditory evoked potentials (P3), with binaural tone burst stimulation at frequencies of 1,000Hz for the frequent stimulus, and of 2,000Hz for the rare stimulus, with an intensity of 80dBHL. Three-hundred stimuli were presented, out of which 240 were frequent and 60 were rare, at a ratio of 80% to 20%, respectively. Two waves were recorded at each scanning, one for the frequent stimulus and one for the rare one. In the end, waves were subtracted to obtain the P300. The P3 wave latency value was considered for the analysis of this potential.^(6)^

Click stimuli were used for the brainstem auditory evoked potential (BAEP), monoaurally presented at 80dBHL, with a rarefaction polarity and at a rate of 19.1 clicks/second. Absolute latencies of waves I, III and V and I-III, III-V and I-V interpeak intervals were recorded. The normalcy criteria were suggested by the equipment manufacturer. Impairments were classified as lower brainstem, upper brainstem and diffuse brainstem impairments.^(7)^

The simplified auditory processing (AP) test was performed at a sound field with the following procedures: sound localization test and memory test for sequential sounds.^(8,9)^

The other behavioral tests were performed in an acoustic booth using verbal and non-verbal stimuli recorded in a CD. The following tests were performed: duration pattern test with pure tone (DPT-PT), free-recall dichotic consonant-vowel test (FR-DCVT), staggered spondaic word test (SSW; quantitative and qualitative analysis), synthetic sentence identification test with competing ipsilateral (SSI-CIM) or contralateral message (SSI-CCM), Random Gap Detection Test (RGDT), percentage speech recognition rate with recording (PSRR) and speech test with white noise (STWN). The criteria of normality used for the AP behavioral test were those described by Pereira.^(10)^

The statistical analysis was performed using Wilcoxon’s test, test of equality of two proportions, confidence interval for the mean with 95% of statistical confidence and p value (level of significance used 0.10 due to small sample).

## RESULTS

The sample consisted of six subjects who sustained severe TBI, five were High School Graduates and one had incomplete Further Education, mean age of 26.7 years.

### Part 1. Electrophysiological testing − BAEP and LLAEP (P3)

**Figure 1:**
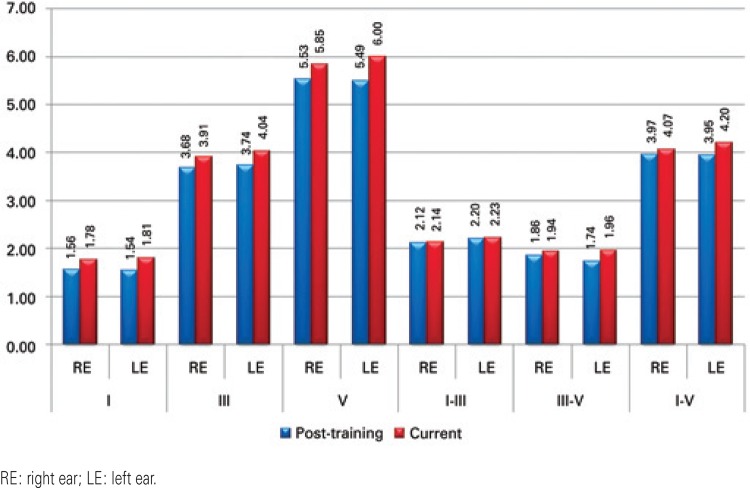
Mean absolute latencies for waves I, III and IV and I-III, III-V and I-V interpeak intervals in the post-training and current tests

The findings showed an increase in absolute latency for all waves and interpeak intervals in the current tests when compared to post-training test results, both for the right and left ear. This increase was statistically significant for wave I, III and V absolute latencies in both ears, as well as for the I-V interpeak interval in the right ear and the III-V and I-V interpeak intervals in the left ear (Figure 1).

**Figure 2:**
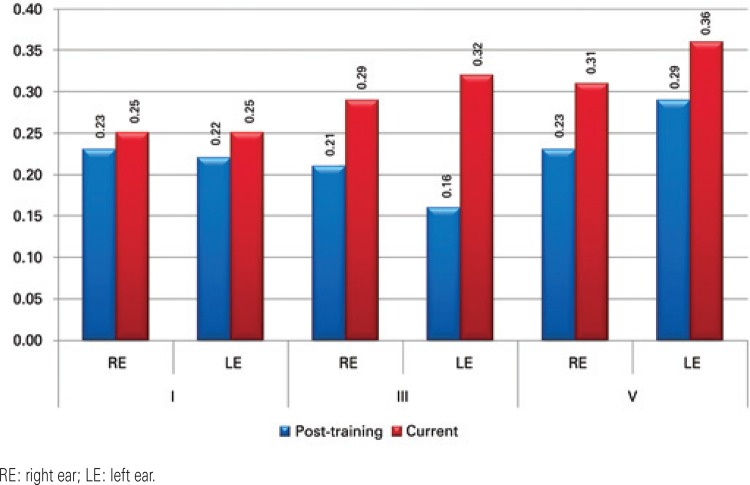
Mean amplitude of waves I, III and V in the post-training and current tests

Right ear BAEP results showed that wave I, III and V amplitudes increased and that the wave V amplitude increase was statistically significant. The left ear BAEP also showed an increase in waves I, III and V amplitude, with a statistically significant increase in wave III amplitude in the current test, when compared to the post-training test (Figure 2).

A decrease was found in P3 latency in both ears, which was statistically significant in the right ear and with a trend towards significance in the left ear (Table 1). Additionally, an increase in P3 amplitude was found in the current test in both ears, with a statistically significant increase in the right ear (Table 2).

**Table 1:** P3 latency (ms) in post-training and current tests

P3 latency	Mean	Median	Standard deviation	p value
Right ear				
Post-training	345.7	340.2	38.7	0.046
Current	302.2	292.0	44.1	
Left ear				
Post-training	335.7	330.7	45.6	0.116
Current	300.3	301.5	38.1	

P3: long latency auditory evoked potential component tested in this study.

**Table 2:** P3 amplitude (mV) in post-training and current tests

P3 amplitude	Mean	Median	Standard deviation	p value
Right ear				
Post-training	5.91	4.34	2.81	0.075
Current	10.19	10.75	3.94	
Left ear				
Post-training	6.07	4.84	2.79	0.249
Current	9.23	10.68	3.22	

P3: long latency auditory evoked potential component tested in this study.

### Part 2. Behavioral testing of auditory processing

The performance in the post-training and current behavioral test of the AP was similar. A statistically significant difference was found only in the performance in the SSW test in the left ear, with worse results in the current test (Table 3). Although the performance in this particular test was worse, the degree of impairment, which is mild, remained the same.

**Table 3:** Descriptive performance measures of the subjects in the post-training and current behavioral test of the auditory processing

Behavioral	Mean (%)	Median (%)	Standard deviation (%)	p value
SL				
Post-training	90.0	90.0	11.0	0.157
Current	96.7	100.0	8.2	
VSM 4 sounds				
Post-training	88.9	100.0	17.2	0.564
Current	83.3	83.3	18.3	
NVSM 4 sounds				
Post-training	83.3	83.3	18.3	0.564
Current	88.9	100.0	17.2	
STWN RE				
Post-training	90.7	90.0	4.8	0.705
Current	89.3	88.0	5.5	
STWN LE				
Post-training	86.7	86.0	5.5	0.581
Current	88.0	88.0	5.7	
SSW RE				
Post-training	96.7	98.8	4.9	0.139
Current	93.9	92.5	2.6	
SSW LE				
Post-training	88.3	88.8	7.5	0.059
Current	85.4	85.0	7.0	
SSI-ICM RE (-10)				
Post-training	83.3	85.0	8.2	0.102
Current	90.0	90.0	6.3	
SSI-ICM LE (-10)				
Post-training	83.3	85.0	8.2	0.157
Current	91.7	95.0	9.8	
DPT				
Post-training	91.1	91.7	8.4	0.144
Current	87.8	90.0	10.3	

SL: sound localization; VSM: verbal sequential memory; NVSM: non-verbal sounds sequential memory; SPWN: speech test with white noise; RE: right ear; LE: left ear; SSW: staggered spondaic word test; SSI-ICM: synthetic sentence identification (with competing ipsilateral message); DPT: duration pattern test.

Regarding the tests that cannot be measured in percent, FR-DCVT results showed that subjects gave less correct responses when compared to post-training tests, however, they still gave more correct responses in the right ear, even though this was not statistically significant, thus showing left hemisphere dominance, as expected. There was a statistically significant increase in the number of incorrect responses in the current test when compared to the post-training test.

In the RGDT, the mean interval in miliseconds of the tested frequencies was shorter in the current test when compared to the post-training test, but the difference was not statistically significant (Table 4).

**Table 4:** Performance in the Random Gap Detection Test in post-training and current tests

RGDT	Mean	Median	Standard deviation	p value
Post-training	10.42	7.5	8.61	0.916
Current	8.50	7.5	2.30	

RGDT: random gap detection test.

## DISCUSSION

Only two studies in the literature have directly approached this subject, so that the results of our study are compared with studies performed in other populations.

### Part 1. Electrophysiological testing – BAEP and LLAEP (P3)

Absolute latency for all waves and interpeak intervals increased in the current test, with a statistically significant difference in absolute latencies of waves I, III and V in both ears, as well as in interpeak intervals I-V in the right ear and interpeak intervals III-V and I-V in the left ear.

A study carried out with normal adults found that BAEP components remained stable for up to three months without any intervention between the first test and the retest, thus confirming the test-retest reliability of the procedures.^(11)^

Researchers assessed subjects who sustained TBI with electrophysiological tests and concluded that this group has lower brainstem impairments at the BAEP, characterized by increased absolute latencies of waves I and III and of the interpeak interval I-III and that these impairments tend to become permanent if subjects do not undergo any specific treatment.^(12)^

In this study, in spite of the increase in latencies of all waves when compared to results achieved after auditory training, interpeak intervals have not changed significantly, remaining within the ranges of normality, thus indicating that the improvement achieved with training was sustained even one year after the intervention.

The amplitudes of the components increased in both ears, with statistically significant increases in the amplitude of wave V in the right ear and of wave III in the left ear. These results showed that a positive neurophysiological change occurred during this period after ACAT, which is a neurophysiological surrogate of neuronal plasticity.

Few studies have used BAEP to follow changes after auditory training, however, one study showed that the method was a sensitive procedure that could measure neuronal plasticity resulting from stimulation after ACAT.^(5)^

The type of potential most indicated to follow neurophysiologial chances resulting from auditory training is LLAEP because neuronal plasticity is more prominent in cortical areas. Several studies were conducted using the P3 component of LLAEP to assess neurophysiological changes that occur after auditory training and they demonstrated improvements in amplitude, latency and even in the morphology of waves after auditory stimulation.^(13,14)^

A study conducted with an adult subject who suffered TBI, with results before and after auditory training, and retesting four months after training, showed that after training amplitude increased in both ears and latency of wave P3 decreased in the right ear. This improvement was not sustained in the post-training retest.^(4)^

These results are not in agreement with those found in this study because latency of component P3 of the LLAEP decreased in both ears. Additionally, there was an increase in the amplitude of P3 in both ears when compared to post-training tests, with a significant increase in the left ear.

Thus, we can state that the neurophysiological changes achieved with training could not only be sustained, but actually improved with the stimulation provided by the environment where subjects live. We should stress the fact that subjects had not undergone any additional treatment after ACAT before the retest.

### Part 2. Behavioral evaluation of the auditory processing

A statistically significant difference was found only in the SSW test results for the left ear, with worse performance in the current test and in the number of incorrect responses in DCVT. Thus, we can state that the change in the neuronal substrate resulting from ACAT which led to a better performance in the behavioral test could be sustained after one year.

Some studies found similar results regarding the maintenance of improvements achieved after auditory training. One study found that most subjects maintained their performance after six months or longer – only 15% could not maintain their performance. According to the authors, children who did not maintain their performance could have been influenced by non-auditory factors during testing (*e.g*. emotional, cognitive, memory or attention issues).^(15)^

A study comparing the performance of children with APD in two tests (Post 1 and Post 2) found that 60% of them maintained the results achieved in Post 1; among the remianing, one improved and three worsened their APD. It is assumed that attention deficit and lack of motivation might have influenced their performance.^(16)^ However, none of these studies were carried out with a population similar to the one that participated in this study, *i.e*., adults with confirmed neurological injury.

Nonetheless, a case study with a post-TBI patient showed that the individual maintained a similar performance in most tests after showing improvement in the post-training tests, and a small decrease in performance in standard frequency and memory tests for verbal sounds, thus demonstrating the maintenance of improvements in behavioral responses four months after the auditory training.^(4)^

Considering that neuronal plasticity in injured individuals is different from the one that occurs in subjects without brain injury, this study and the comparison with other studies in different populations showed that ACAT can effectively result in neuronal plasticity by stimulating individuals after TBI and that the benefits achieved with training were sustained.

Many patients with severe traumatic brain injury undergo many surgical procedures and stay a long time in hospital. It is often the case that hearing complaints go unnoticed considering the overall severity of the condition. Despite the small sample, this study had strict inclusion criteria and is important because it could demonstrate that auditory neuronal *déficits* can affect social, academic and professional reintegration of these mostly young patients. The auditory stimulation provided by ACAT can be a rehabilitation alternative for these individuals, improving their quality of life and expanding the roles they may have in society.

During the retesting procedures subjects reported a great improvement in their day-to-day activities and better attention and memory capacity, thus facilitating their social reintegration, as well as the return to their professional life and a pleasant life with family and friends.

Among the limitations of this study were the small sample resulting from strict inclusion criteria; the limited number of studies involving a similar population, which made it difficult to discuss results; and the non-validation of the subjective improvement reported by patients through questionnaires.

All patients participated in a care protocol that included auditory training. However, changes found in the evaluations used the subjects as their own controls, which is a procedure used in studies that use therapeutical interventions.

Further studies should be conducted with a similar population including a comparison with a control, randomized group and with a larger sample. This study was carried out with a small sample and with no control group due to the strict inclusion criteria.

## CONCLUSION

The acoustically controled auditory training may have been efficient in the long term, since better results were found for latency and amplitude in the electrophysiological tests, in addition to stable results in the behavioral measures one year after the training.
