# Supplementary material for: Behavioral and electrophysiological auditory processing measures in traumatic brain injury after acoustically controlled auditory training: a long-term study
Source: Einstein (Sao Paulo). 2015 Oct-Dec;13(4):535–40. doi: 10.1590/S1679-45082015AO3379 (PMC4878627; doi:10.1590/S1679-45082015AO3379)
Supplement: Translation

# Translation: Medidas comportamentais e eletrofisiológicas do processamento auditivo em traumatismo craniencefálico após treinamento auditivo acusticamente controlado: estudo de longo prazo

**Authors:** Carolina Calsolari Figueiredo, Adriana Neves de Andrade, Andréa Tortosa Marangoni-Castan, Daniela Gil, Italo Capraro Suriano

**Affiliations:** 1 Universidade Federal de São Paulo, São Paulo, SP, Brasil.

**Keywords:** Plasticidade neuronal, Transtornos da percepção auditiva, Aprendizagem, Estimulação acústica, Traumatismos encefálicos/complicações

## Abstract

**Objetivo:**

Investigar a eficácia de longo prazo do treinamento auditivo acusticamente controlado em indivíduos adultos após traumatismo craniencefálico.

**Métodos:**

Foram estudados seis indivíduos audiologicamente normais com idades entre 20 e 37 anos, que sofreram traumatismo craniencefálico grave com lesão axional difusa, submetidos a um programa de treinamento auditivo acusticamente controlado aproximadamente um ano antes. Foram comparados os resultados obtidos nas avaliações comportamental e eletrofisiológica do processamento auditivo imediatamente após o treinamento auditivo acusticamente controlado e a reavaliação, um ano após.

**Resultados:**

Quanto ao potencial de tronco encefálico, observou-se aumento da latência absoluta de todas as ondas e os intervalos interpicos, na comparação entre as avaliações, bilateralmente, bem como aumento da amplitude de todas as ondas, sendo a onda V estatisticamente significativa, para a orelha direita e a onda III para a orelha esquerda. Quanto ao P3, observaram-se diminuição da latência e aumento da amplitude do P3 na avaliação atual em ambas as orelhas. A avaliação comportamental atual evidenciou desempenho semelhante nas duas avaliações, exceto no teste dicótico de dissílabos alternados na orelha esquerda e a quantidade de erros no teste dicótico consoante-vogal.

**Conclusão:**

O treinamento auditivo acusticamente controlado mostrou-se eficaz em longo prazo, uma vez que foram observados resultados melhores de latência e amplitude na avaliação eletrofisiológica, bem como estabilidade das medidas comportamentais após um ano de treinamento.

## INTRODUÇÃO

O traumatismo craniencefálico (TCE) é definido como qualquer agressão traumática que tenha como consequência lesão anatômica ou comprometimento funcional das meninges, encéfalo ou de seus vasos.^(1)^

A deformação do cérebro pela aceleração e desaceleração causa lesões primárias que podem afetar o substrato neural responsável pela audição, podendo gerar *déficit *auditivos centrais, envolvendo áreas auditivas corticais e subcorticais. Assim, indivíduos que sofrem TCE podem apresentar distúrbio do processamento auditivo (DPA), que podem ser identificados com avaliações eletrofisiológicas e comportamentais.^(2)^

O treinamento auditivo acusticamente controlado (TAAC) é um conjunto de condições e/ou tarefas acústicas controladas e modeladas da maneira desejada, visando maximizar os efeitos da plasticidade do sistema nervoso central e outros sistemas relacionados, de tal forma que suas bases neurais e comportamentos auditivos sejam alterados de maneira positiva.^(2,3)^

Com o TAAC, é possível habilitar ou reabilitar as habilidades auditivas que auxiliam os processamentos linguístico e fonêmico necessários para a compreensão da fala e para a efetividade das funções comunicativas.

Foram encontrados apenas dois estudos na literatura realizados com indivíduos que sofreram TCE submetidos ao TAAC, e ambos revelaram que o treinamento foi eficiente em provocar a plasticidade neuronal por meio de estimulação auditiva.^(4,5)^ No entanto, a reavaliação foi realizada logo após o término das sessões de estimulação, sem acompanhamento longitudinal dos pacientes.

## OBJETIVO

Investigar a eficácia a longo prazo do treinamento auditivo acusticamente controlado em indivíduos adultos após traumatismo craniencefálico.

## MÉTODOS

Pesquisa realizada nos laboratórios de processamento auditivo e de eletrofisiologia da Disciplina de Distúrbios de Audição da Universidade Federal de São Paulo e aprovada pelo Comitê de Ética em Pesquisa da mesma universidade, sob número 0389/10. Todos os indivíduos assinaram o Termo de Consentimento Livre e Esclarecido.

Os seis participantes eram do sexo masculino, com idade entre 20 e 37 anos. Todos apresentavam lesão axional difusa, sendo que dois indivíduos não apresentavam lesão focal associada, dois apresentavam hematoma subdural, um apresentava contusão temporal, e um apresentava hematoma subdural e contusão temporal associados.

Os critérios de inclusão adotados foram apresentar TCE fechado grave (escala Glasgow de 3 a 8 na admissão hospitalar), permanência em coma induzido, lesão axonal difusa, tempo entre a lesão e a participação no estudo de 3 a 24 meses, faixa etária entre 18 e 50 anos, preferência manual direita, Ensino Médio completo, limiares auditivos dentro dos padrões da normalidade entre 250 e 4.000Hz.

Todos os indivíduos foram submetidos a um programa de TAAC aproximadamente um ano antes. Foram comparados os resultados obtidos das avaliações comportamentais e eletrofisiológicas do processamento auditivo imediatamente após o TAAC e após 1 ano da realização do mesmo.

A captação dos potenciais evocados auditivos (PEA) foi iniciada pelo potencial evocado auditivo de longa latência (P3), com estímulos auditivos binaurais tipo *tone burst* com frequências de 1.000Hz para o estímulo frequente, e 2.000Hz para o estímulo raro, com intensidade de 80dBNA. Foram apresentados 300 estímulos, sendo 240 frequentes e 60 raros, numa proporção de 80% para 20%, respectivamente. A cada varredura, foram gravadas duas ondas, uma para o estímulo frequente e outra para o raro. Ao final, as ondas foram subtraídas para obtenção do P300. Para a análise desse potencial, foi considerado o valor de latência da onda P3.^(6)^

Para o potencial evocado auditivo de tronco encefálico (PEATE), foram utilizados estímulos do tipo clique, apresentados monoauralmente a 80dBNA, com polaridade rarefeita e velocidade de 19,1 cliques/segundo. Foram registradas as latências absolutas das ondas I, III, e V e dos intervalos interpicos I-III, III-V e I-V. Os critérios de normalidade utilizados foram os sugeridos pelo fabricante do equipamento. As alterações foram classificadas em tronco encefálico baixo, tronco encefálico alto e alteração difusa de tronco encefálico.^(7)^

A avaliação simplificada do processamento auditivo (PA) foi realizada em campo sonoro com os seguintes procedimentos: teste de localização sonora e teste de memória para sons em sequência.^(8,9)^

Os demais testes comportamentais foram realizados em cabina acústica, utilizando-se estímulos verbais e não verbais gravados em CD. Foram realizados os seguintes testes: teste padrão de duração com tom puro (TPD-TP), teste dicótico consoante-vogal etapa de atenção livre (TDCV-AL), teste dicótico de dissílabos alternados (SSW; *staggered spondaic word*; análise quantitativa e qualitativa), teste de identificação de sentenças sintéticas com mensagem competitiva ipsilateral (SSI-MCI) ou contralateral (SSI-MCC), Teste de Fusão Auditiva Randomizado (RGDT - *Random Gap Detection Test*), índice porcentual de reconhecimento de fala com gravação (IPRF) e teste de fala com ruído branco (TFRB). Os critérios de normalidade utilizados para avaliação comportamental do PA foram os descritos por Pereira.^(10)^

A análise estatística foi realizada utilizando o teste de Wilcoxon, teste de igualdade de duas proporções, intervalo de confiança para média com 95% de confiança estatística e valor de p (nível de significância adotado de 0,10, devido à baixa amostragem).

## RESULTADOS

A amostra foi constituída por seis indivíduos, cinco com Ensino Médio completo e um Ensino Superior incompleto, com média de idade de 26,7 anos, que sofreram TCE grave.

### Parte 1. Avaliação eletrofisiológica − PEATE e PEALL (P3)

Os achados evidenciaram aumento da latência absoluta de todas as ondas e dos intervalos interpicos na avaliação atual, em comparação aos resultados obtidos após o treinamento, tanto na orelha direita quanto na orelha esquerda. Esse aumento foi estatisticamente significante para as latências absolutas das ondas I, III e V em ambas as orelhas, bem como o intervalo interpico I-V na orelha direita e os intervalos interpicos III-V e I-V na orelha esquerda (Figura 1).

**Figura 1:** Latências absolutas médias das ondas I, III e V e dos intervalos interpicos I-III, III-V e I-V, nas avaliações após treinamento e atual

Em relação à amplitude das ondas I, III e V do PEATE na orelha direita, observou-se que todas as amplitudes aumentaram, sendo o aumento da amplitude da onda V estatisticamente significante na avaliação. Na orelha esquerda também houve aumento da amplitude das ondas I, III e V do PEATE com aumento estatisticamente significante na amplitude da onda III na avaliação atual, quando comparada à avaliação após treinamento (Figura 2).

**Figura 2:** Amplitude média das ondas I, III e V nas avaliações após treinamento e atual

Houve diminuição da latência do P3 em ambas as orelhas, estatisticamente significante à orelha direita e com tendência à significância à orelha esquerda (Tabela 1). Também houve aumento da amplitude do P3 na avaliação atual em ambas as orelhas, sendo que esse aumento foi estatisticamente significante à orelha direita (Tabela 2).

**Tabela 1:** Latência (ms) do P3 nas avaliações após treinamento e atual

P3 latência	Média	Mediana	Desvio padrão	Valor de p
Orelha direita				
Após treinamento	345,7	340,2	38,7	0,046
Atual	302,2	292,0	44,1	
Orelha esquerda				
Após treinamento	335,7	330,7	45,6	0,116
Atual	300,3	301,5	38,1	

P3: componente do potencial evocado auditivo de longa latência analisado neste estudo.

**Tabela 2:** Amplitude (mV) do P3 nas avaliações após treinamento e atual

P3 amplitude	Média	Mediana	Desvio padrão	Valor de p
Orelha direita				
Após treinamento	5,91	4,34	2,81	0,075
Atual	10,19	10,75	3,94	
Orelha esquerda				
Após treinamento	6,07	4,84	2,79	0,249
Atual	9,23	10,68	3,22	

P3: componente do potencial evocado auditivo de longa latência analisado neste estudo.

### Parte 2. Avaliação comportamental do processamento auditivo

Na reavaliação comportamental do PA, o desempenho mostrou-se semelhante quando comparadas as duas situações. Houve diferença estatisticamente significante apenas entre os desempenhos no teste SSW na orelha esquerda, com piora dos resultados na situação de avaliação atual (Tabela 3). Importante ressaltar que, apesar da piora no desempenho, houve manutenção do grau da alteração, classificado como leve.

**Tabela 3:** Medidas descritivas do desempenho dos indivíduos na avaliação comportamental do processamento auditivo após treinamento e atual

Comportamental	Média (%)	Mediana (%)	Desvio padrão (%)	Valor de p
LS				
Após treinamento	90,0	90,0	11,0	0,157
Atual	96,7	100,0	8,2	
MSV 4 sons				
Após treinamento	88,9	100,0	17,2	0,564
Atual	83,3	83,3	18,3	
MSNV 4 sons				
Após treinamento	83,3	83,3	18,3	0,564
Atual	88,9	100,0	17,2	
TFRB OD				
Após treinamento	90,7	90,0	4,8	0,705
Atual	89,3	88,0	5,5	
TFBR OE				
Após treinamento	86,7	86,0	5,5	0,581
Atual	88,0	88,0	5,7	
SSW OD				
Após treinamento	96,7	98,8	4,9	0,139
Atual	93,9	92,5	2,6	
SSW OE				
Após treinamento	88,3	88,8	7,5	0,059
Atual	85,4	85,0	7,0	
SSI-MCI OD (-10)				
Após treinamento	83,3	85,0	8,2	0,102
Atual	90,0	90,0	6,3	
SSI-MCI OE (-10)				
Após treinamento	83,3	85,0	8,2	0,157
Atual	91,7	95,0	9,8	
TPD				
Após treinamento	91,1	91,7	8,4	0,144
Atual	87,8	90,0	10,3	

LS: localização sonora; MSV: memória sequencial verbal; MSNV: memória sequencial de sons não verbais; TFRB: teste de fala com ruído branco; OD: orelha direita; OE: orelha esquerda; SSW: teste dicótico de dissílabos alternados; SSI-MCI: identificação de sentenças sintéticas (mensagem competitiva ipsilateral); TPD: teste padrão de duração.

Quanto aos testes que não podem ser mensurados em porcentagem, foi possível observar que no TDCV-AL os indivíduos apresentaram menor número de acertos em comparação à avaliação após treinamento, porém mantiveram mais acertos na orelha direita, mesmo que sem significância estatística, demonstrando dominância hemisférica esquerda, como esperado. Quanto aos erros, houve aumento estatisticamente significante no número de erros na avaliação atual, quando comparada à avaliação após treinamento.

No teste RGDT, observou-se que o intervalo médio em milissegundos das frequências testadas foi menor na avaliação atual quando comparado com a avaliação após treinamento, sem significância estatística (Tabela 4).

**Tabela 4:** Desempenho no Teste de Fusão Auditiva Randomizado nas avaliações após treinamento e na atual

RGDT	Média	Mediana	Desvio padrão	Valor de p
Após treinamento	10,42	7,5	8,61	0,916
Atual	8,50	7,5	2,30	

RGDT: Teste de Fusão Auditiva Randomizado.

## DISCUSSÃO

Há apenas dois estudos na literatura que abordam diretamente o tema proposto, de modo que os resultados aqui também são discutidos com estudos realizados em outras populações.

### Parte 1. Avaliação eletrofisiológica – PEATE e PEALL (P3)

Houve aumento da latência absoluta de todas as ondas e dos intervalos interpicos na avaliação atual, sendo estatisticamente significante para as latências absolutas das ondas I, III e V em ambas as orelhas, bem como intervalo interpicos I-V na orelha direita e os intervalos interpicos III-V e I-V na orelha esquerda.

Em estudo realizado com adultos normais, verificou-se estabilidade dos componentes do PEATE no período de até 3 meses, sem qualquer intervenção entre a avaliação e a reavaliação, afirmando a confiabilidade teste-reteste dos procedimentos.^(11)^

Pesquisadores avaliaram indivíduos que sofreram TCE por meio de testes eletrofisiológicos e concluíram que esse grupo apresentou alteração de tronco baixo no PEATE, caracterizado por aumento das latências absolutas das ondas I e III e do intervalo interpico I-III, e que essas alterações tendem a ser permanentes, caso não haja intervenção terapêutica específica.^(12)^

Neste estudo, apesar do aumento das latências de todas as ondas em relação à avaliação após o treinamento auditivo, os intervalos interpicos pouco variaram, permanecendo dentro da normalidade e indicando que a melhora obtida após o treinamento permaneceu mesmo um ano depois da intervenção.

As amplitudes dos componentes aumentaram em ambas as orelhas, sendo estatisticamente significantes o aumento da amplitude da onda V na orelha direita e o aumento da onda III na orelha esquerda. Esses resultados mostraram que houve mudança neurofisiológica positiva neste período após o TAAC, evidenciando um correlato neurofisiológico da plasticidade neural.

Existem poucos trabalhos que tenham utilizado o PEATE para monitorar as modificações após treinamento auditivo, contudo um estudo evidenciou que o PEATE mostrou-se um procedimento sensível para mensurar a plasticidade neuronal provocada pela estimulação após TAAC.^(5)^

O tipo de potencial mais indicado para monitorar as mudanças neurofisiológicas provocadas pelo treinamento auditivo é o PEALL, pois a plasticidade neuronal é maior nas regiões corticais. Diversos estudos já foram realizados utilizando o componente P3 do PEALL, para avaliar as mudanças neurofisiológicas que ocorrem após o treinamento auditivo e os mesmos demonstraram melhora na amplitude, latência e até mesmo na morfologia das ondas após o período de estimulação auditiva.^(13,14)^

Estudo realizado com um indivíduo adulto que sofreu TCE, com resultados pré e pós-treinamento auditivo, e reavaliação 4 meses após o treinamento, evidenciou que, depois do treinamento, houve aumento da amplitude em ambas as orelhas e diminuição da latência na orelha direita da onda P3. Na reavaliação, após o treinamento, essa melhora não se manteve.^(4)^

Esses resultados não corroboram os encontrados no presente estudo, pois houve diminuição da latência do componente P3 do PEALL em ambas as orelhas. Foi possível verificar também que aumento da amplitude do P3 em ambas as orelhas, quando comparadas com as avaliações, sendo significante na orelha esquerda.

Dessa forma, pode-se afirmar que as mudanças neurofisiológicas obtidas com o treinamento não apenas se mantiveram, como melhoraram em presença da estimulação do meio em que o indivíduo vive, salientando que, após o TAAC, não foi realizado qualquer outro procedimento terapêutico nos indivíduos, até o momento da reavaliação.

### Parte 2. Avaliação comportamental do processamento auditivo

Houve diferença estatisticamente significante apenas entre os desempenhos no teste SSW na orelha esquerda, com piora dos resultados na situação de avaliação atual e no número de erros cometidos no TDCV-AL. Com isso, é possível afirmar que a modificação no substrato neural proporcionada pelo TAAC, que resultou em melhora no desempenho nos testes comportamentais, manteve-se após o período de 1 ano.

Alguns estudos obtiveram resultados semelhantes em relação à manutenção das melhoras obtidas após treinamento auditivo. Um estudo observou que a maioria dos sujeitos estudados manteve seu desempenho após 6 meses ou mais − apenas 15% que não mantiveram o desempenho. Para os autores, as crianças que não mantiveram seu desempenho podem ter sofrido a interferência de fatores não auditivos na avaliação (por exemplo: alterações emocionais, cognitivas, de memória ou de atenção).^(15)^

Estudo que comparou o desempenho de crianças com DPA em duas avaliações (Após 1 e Após 2), em relação ao grau do DPA, registrou que 60% das crianças mantiveram os resultados obtidos no Após 1; entre as demais, uma apresentou melhora e três apresentaram piora do grau. Para esses indivíduos, considerou-se que a dificuldade de atenção e a desmotivação podem ter influenciado no desempenho.^(16)^ No entanto, nenhum desses estudos aqui mencionados foram realizados com população semelhante à presente estudo, ou seja, adultos com lesão neurológica confirmada.

Porém, em estudo de caso com paciente pós-TCE, o paciente manteve desempenho semelhante na maioria dos testes nos quais tinha mostrado melhora da avaliação após o treinamento, além de ligeira queda no desempenho nos testes de padrão de frequência e memória para sons verbais, evidenciando manutenção das melhoras para as respostas comportamentais 4 meses após o treinamento auditivo.^(4)^

Considerando que a plasticidade neuronal em indivíduos lesados ocorre de forma diferente daqueles sujeitos sem lesão cerebral, a realização deste estudo e sua comparação com outros estudos realizados com populações diferentes evidenciam que o TAAC mostrou-se eficiente em provocar a plasticidade neuronal, por meio de estimulação nos indivíduos após TCE, e que os benefícios obtidos com o treinamento se mantiveram nesses indivíduos.

Muitos dos pacientes acometidos por traumas cranianos graves passam por inúmeras intervenções cirúrgicas e por longos períodos de internação. Muitas vezes, as queixas auditivas acabam não sendo valorizadas, diante da gravidade geral dos acometimentos. Este estudo, apesar de contar com uma amostra pequena, devido à rigidez dos critérios de inclusão, mostra-se importante por demonstrar os *déficits* neurais auditivos que podem comprometer a reinserção social, acadêmica e profissional destes pacientes, que, em sua maior parte, são jovens. A estimulação auditiva promovida pelo TAAC pode ser uma alternativa de reabilitação para esses indivíduos, promovendo melhora da qualidade de vida e das possibilidades de atuação na sociedade.

Os indivíduos, durante a reavaliação, relataram grande melhora no dia a dia, e melhor capacidade de atenção e memória, facilitando a reinserção social, bem como a retomada da vida profissional e do convívio agradável com seus familiares e amigos.

É importante ressaltar que foram limitações deste estudo a baixa amostragem, resultado da rigidez dos critérios de inclusão; os poucos estudos realizados com população semelhante à estudada, que dificultou a discussão dos resultados obtidos; e a não validação da melhora subjetiva relata pelos pacientes, por meio de questionários.

Todos os pacientes foram inseridos no protocolo de atendimento que incluía a realização de treinamento auditivo. No entanto, reforça-se que as modificações verificadas nas avaliações utilizaram o indivíduo como seu próprio controle, conduta que é indicada em estudos que utilizam intervenções terapêuticas.

Devem ser realizados novos estudos com população semelhante, que incluam a comparação com um grupo controle, randomizado e com maior amostragem. Pela rigidez dos critérios de inclusão, este estudo foi realizado com baixa amostragem e sem grupo controle.

## CONCLUSÃO

O treinamento auditivo acusticamente controlado pode ter sido eficaz, em longo prazo, uma vez que foram observados resultados melhores de latência e amplitude na avaliação eletrofisiológica, bem como estabilidade das medidas comportamentais após um ano do treinamento.
